# False aneurysm of the superficial palmar arch in a child: a case report

**DOI:** 10.4076/1757-1626-2-7985

**Published:** 2009-07-24

**Authors:** Akio Sakamoto, Ken Arai

**Affiliations:** 1 Department of Orthopaedic Surgery, Graduate School of Medical Sciences, Kyushu University3-1-1 Maidashi, Fukuoka, 812-8582, FukuokaJapan; 2 Department of Orthopaedic Surgery, Iizuka HospitalIizuka, 820-8505, FukuokaJapan

## Abstract

**Introduction:**

False aneurysm of the arteries of the hand is extremely rare.

**Case presentation:**

We report a case of false aneurysm of the superficial palmar arch in a 4-year-old boy following injury with a piece of glass. Stereoscopic magnetic resonance angiography depicted the lesion effectively. The aneurysm was resected, and the vascular reconstruction was performed with end-to-end anastomosis using microsurgical techniques.

**Conclusions:**

Follow-up care is necessary after a hand injury in order to properly diagnose any lesion that may be present and treat it successfully.

## Introduction

A false aneurysm lacks the normal architecture of the arterial wall, while a true aneurysm is a dilation of the artery in which the arterial wall retains its normal architecture. A false aneurysm forms a cystic structure with a thrombus after disruption of the arterial wall [1]. A false aneurysm in the hand is rare. When it occurs, the digital artery is the usual site [2-5]. The superficial palmar arch is an extremely rare site for a false aneurysm. Because of the rarity of this kind of lesion in the hand, initial misdiagnosis is common.

We identified four cases which specify false aneurysms of the superficial palmar arch. Most cases were adults, including a 50-year-old male [6], a 37-year-old male [7], and a 38-year-old female [8]. The other case was a 5-year-old girl [1]. According to the authors, the case in the child was the first reported in the literature in English.

## Case presentation

**Figure 1.**
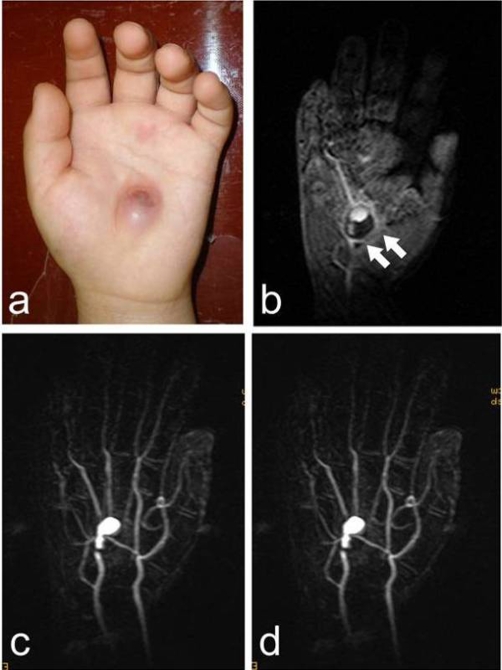
A pulsated mass in the palm can be seen **(a)**. The T1-weighted MRI image shows a low-signal intensity area suggesting a thrombus within the lesion (arrows) **(b)**. A stereoscopic MRA image shows an aneurysm in the superficial palmar arch **(c, d)**.

A 4-year-old Japanese boy cut the palm of his hand with a piece of glass. The wound was sutured at a hospital nearby. The wound was only 1 cm long, and the bleeding was stopped without any microscopic procedure. Ten days after the initial injury, swelling appeared progressively in the palm of the hand near the site of the scar. The boy was referred to our institution. Clinical examination showed a palpated mass 2 cm in diameter in the palm of the hand (Figure 1a), as well as a cystic lesion with thrombus (Figure 1b). Stereoscopic magnetic resonance angiography (MRA) suggested that the cyst was an aneurysm located at the superficial palmar arch (Figures 1c, 1d).

**Figure 2.**
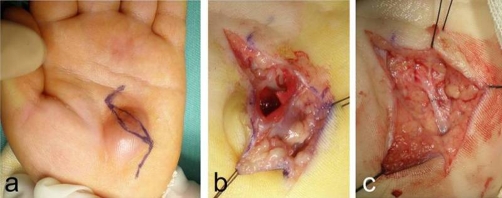
The incision was designed (blue marker) in order to resect the skin above the lesion **(a)**. The false aneurysm continuing to the superficial palmar branch can be seen **(b)**. The false aneurysm was removed en bloc, and vascular reconstruction via end-to-end anastomosis was performed **(c)**.

Surgical exploration showed a 1.5 cm aneurysm involving the superficial palmar arch. The thinned skin above the aneurysm was resected. The superficial palmar arch was ligated at both sides of the aneurysm, and the mass was removed en bloc. Vascular reconstruction was performed with end-to-end anastomosis using microsurgical techniques (Figure 2). No anticoagulant medications were applied either during or post-surgery locally or generally. Histological examination confirmed the diagnosis of false aneurysm, based on the fact that the aneurysmal cyst lacked whole components of the vessel (data not shown). In the duration of a 2-year follow up, no recurrence nor peripheral vascular problem has been seen.

## Discussion

A true aneurysm is formed due to a weakening or ballooning of the vessel wall. In the case of a false aneurysm, the arterial wall is partially or completely disrupted, resulting in haemorrhage into the surrounding tissue. Eventually, the wall of the haematoma is replaced by fibrous tissue and a false aneurysm forms [1]. False aneurysms are suspected when a pulsated mass arises progressively [1]. False aneurysms in the hand are generally related to penetrating trauma [8]. On the other hand, repetitive microtrauma can cause a false aneurysm to develop. Interestingly, a case of false aneurysm of the digital artery of the thumb associated with the sport of baseball has been reported [5].

Angiography is very useful and provides information on localization of the aneurysm and collateral circulation [9]. However, angiography appears to be controversial because of possible complications, such as the risk of distal embolization [1]. MRA is useful and non-invasive because it does not need any contrast medium [8]. Moreover, as in the current case, stereoscopic MRA provides effective information regarding the location. As for treatment, surgical treatment comprising resection with arterial ligature [10] or arterial reconstruction [2,3,11,12] is recommended for the lesion. It has been assumed that restoration of the normal blood flow is always preferable, especially in children [1]. Successful reconstruction using end-to-end anastomosis has been reported in a case of false aneurysm in the digital artery similar to the current case [5].

## Conclusions

In conclusion, false aneurysm of the hand is extremely rare, especially in a child. We experienced a successful outcome with treatment comprising end-to-end anastomosis following resection of a false aneurysm in the superficial palmar arch. It would seem that the preferred treatment is surgical resection of a false aneurysm followed by arterial reconstruction.

## Abbreviation

MRA: magnetic resonance angiography
